# Lupus Recipe inhibits cGVHD‐induced lupus nephritis in mice and promote renal LC3‐associated autophagy

**DOI:** 10.1002/iid3.815

**Published:** 2023-03-17

**Authors:** Ruihua Liu, Xuan Huang, Hongjian Ye, Haishan Wu, Jing Guo, Yuan Peng, Meiju Wu, Jinjin Fan, Xiao Yang

**Affiliations:** ^1^ Department of Nephrology, The First Affiliated Hospital Sun Yat‐sen University Guangzhou China; ^2^ NHC Key Laboratory of Clinical Nephrology (Sun Yat‐Sen University) and Guangdong Provincial Key Laboratory of Nephrology Guangzhou China

**Keywords:** cytokines, inflammation, phagocytosis, systemic lupus erythematosus

## Abstract

Lupus nephritis (LN) is one of the most severe manifestations of systemic lupus erythematosus (SLE). The chronic graft versus host disease (cGVHD) mouse model is a well‐established model of SLE. LC3‐associated autophagy plays a critical role in extracellular particle clearance, including pathogens and apoptotic cells. Lupus Recipe (LR) is a Chinese herbal compound that has been proven to be effective in treating SLE. In the study, we investigated the protective effects of LR or LR combined with prednisone on cGVHD mouse model and LC3‐associated autophagy in the kidney. The mice were subjected to six groups. The LR treatment group received LR at the dosage of 1.15 and 2.3 g/kg/day, respectively. The corticosteroid treatment group received prednisone at a dosage of 5 mg/kg/day. The combination treatment group received LR at a dosage of 2.3 g/kg/day, and prednisone at 2.5 mg/kg/day. LR treatment reduced proteinuria and serum triglyceride levels, as well as spleen weight. LR also alleviated pathologic damage and immunoglobulin G deposition in the kidney. LR combined with a low dose of prednisone significantly improved kidney function and decreased serum triglyceride, total cholesterol, and spleen weight. In addition, combination treatment relieved kidney injury more effectively than LR alone. Western blot revealed that LR treatment or LR combined with prednisone increased the LC3‐associated autophagy protein of Rubicon and Nox2, as well as LC3I levels in the kidney tissues. In conclusion, LR inhibited the manifestation of cGVHD‐induced LN, which may attribute to the increased levels of LC3‐associated autophagy.

## Graphical abstract

The Chinese Herbal Compound Lupus Recipe (LR) could effectively ameliorate the inflammatory damage of lupus mice. The combination treatment of LR and low dose prednisone could better inhibit the progression of lupus. The potential mechanism may involve in the regulation of LC3‐associated autophagy and immune‐inflammatory response.

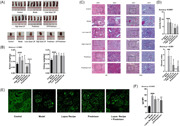

## 1 INTRODUCTION

Lupus nephritis (LN) is one of the most severe complications of systemic lupus erythematosus (SLE) and a critical cause of end‐stage renal disease (ESRD) and inferior patient survival.^1^ With the widespread use of corticosteroid therapy, and immunosuppressive agents or newly emerging biological agents,^2^ patient survival improved significantly. However, many observational studies had confirmed that with the cumulative dose of corticosteroid increasing, the occurrence of corticosteroid‐attributable damages would increase significantly.^3^, ^4^, ^5^ These damages include osteonecrosis, cataracts, diabetes, and bone fractures et al, which might affect patients' quality of life and compliance.^5^ A combination of corticosteroids with immunosuppressants, hydroxychloroquine, or Traditional Chinese Medicine might provide a viable option for the limitation.

The Jiedu Huoxue Treatment Method, represented by Lupus Recipe (LR), was invented to treat SLE with noxious heat and blood stasis syndrome. The LR is made up of seven herbs which include *Hedyotis diffusa* Willd., *Scutellaria barbata* D. Don., *Lithospermum erythrorhizon Sieb*.et Zucc, *Salvia miltiorrhiza* Bunge., *leonurus japonicus* Houtt., *Radix Rehmanniae*, and *Scorpion*.^6^ We have previously demonstrated that a combination of LR and immunosuppressants could significantly reduce disease flares and side effects in LN patients compared with immunosuppressants alone.^7^ Subsequently, we did a series of studies to clarify the potential mechanism of LR treating SLE. We found that LR treatment could significantly inhibit the secretion of interleukin (IL)‐6 and IL‐10 in renal tissue and lymphocytes, restrained the expression of renal C‐C chemokine ligand 5 (RANTES), and ameliorated the glomerulosclerosis, renal tubular injury, and interstitial inflammation of lupus mice.^8^, ^9^, ^10^ Additionally, in vitro experiments showed that serum containing LR could significantly enhance the expression of peroxisome proliferator‐activated receptor γ (PPARγ) while suppressing the expression of CD40 and RANTES in renal tubular epithelial cells.^11^ Moreover, another study found that the LR granule could significantly improve the renal function of MRL/lymphoproliferation (MRL/lpr) mice, and the mechanism might be through the downregulation of the nuclear factor‐kappa B (NF‐κB) signaling pathway.^6^ These results indicate that LR could alleviate the renal injury of lupus via immunoregulation and anti‐inflammatory effect. Nevertheless, whether the combination therapy of LR with a low dose of corticosteroid is effective and the potential mechanism remains unclear.

Canonical autophagy is considered to be a critical mechanism for the degradation of cellular components and dysfunctional organelles to maintain cellular homeostasis.^12^ Canonical autophagy can be divided into three categories of macro‐, micro‐, and chaperone‐mediated autophagy. These processes respond to cellular stress and nutrient status.^12^ Noncanonical autophagy is a newly discovered pathway that requires some of the autophagy components but functions distinctive from canonical autophagy. The noncanonical autophagy is also called microtubule‐associated proteins 1A/1B light chain 3 (LC3)‐associated autophagy (LAP), characterized by LC3 conjugated to the phagosome membrane.^13^ NADPH‐oxidase‐2 (NOX2) and rubicon (RUBCN) are the two key modulators of LAP. LAP plays a critical role in extracellular particle clearance, including pathogens and apoptotic cells. The intact function of LAP facilitates a rapid form of phagocytosis, mostly by macrophages, and produces an “immunologically silent” clearance of the apoptosis cells.^14^ Evidence showed that mice lacking *Nox2* or *Rubcn* gene but not canonical autophagy‐related gene displayed defects in apoptotic cell clearance and developed into SLE‐like phenomena.^13^ Another study found that mice with genetic silencing of *Wdfy4* in B cells could reduce the severity of SLE phenotypes by modulating B cell survival via noncanonical autophagy.^15^ These studies indicate that noncanonical autophagy might be a therapeutic target for SLE and LN. We assumed that the LR treatment may exert an anti‐inflammatory effect on LN by regulating the LAP signaling pathway. In this study, we intend to further clarify the potential mechanism of LR treating LN, and the effectiveness of combination therapy of LR with a low dose of corticosteroid.

## 2 METHODS

### 2.1 Mice

The 6 to 8‐week‐old female (C57BL/6×DBA/2) B6D2F1 mice and DBA/2 mice were purchased from Beijing Vital River. This study was carried out in the Experimental Animal Center of Sun Yat‐Sen University (certification number: SYXK‐2019‐0209), and followed the recommendations of Sun Yat‐Sen University for the Use and Care of Animals (SYSU‐IACUC‐2019‐000300).

### 2.2 Reagents and antibodies

Blood urea nitrogen (BUN), serum triglyceride, and serum total cholesterol were performed with a biochemical analyzer (Roche, COBAS C311). Serum creatinine (Scr) was examined with Creatinine Assay Kit (sarcosine oxidase) purchased from Nanjing Jian Cheng Bioengineering Institute (C011‐2‐1). Mouse Anti‐dsDNA Ig's (Total A + G + M) ELISA Kits (Catalog # 5110) were provided by Alpha Diagnostic International. Immunoglobulin G (IgG) (Total) Mouse Uncoated ELISA Kits (Catalog # 88‐50400‐88) were purchased from Invitrogen. Anti‐Rubicon/Baron antibody (ab156052), anti‐NOX2/gp91phox antibody (ab80508), and Goat‐anti‐Mouse IgG H&L (Alexa Fluor® 488) (ab150113) were from Abcam. LC3A/B (D3U4C) XP® Rabbit mAb (#12741S), and β‐Actin (8H10D10) Mouse mAb (#3700) were from Cell Signaling Technology. 10 × RIPA Lysis Buffer (20‐188, Merck Millipore) was used for Western Blot. Rubicon, Nox2, and LC3 proteins were normalized to β‐actin. Urine was collected by properly pressing on the bladder of mice at a single time point and urine protein was measured semiquantitatively using urine dipsticks (Global Biotech Co., Ltd).^16^

### 2.3 Murine model of chronic graft versus host disease (cGVHD)

The female DBA/2 mice were euthanized, and single‐cell suspensions were prepared from the spleen and thymus. After being filtered through a 40‐μm sterile mesh, lysed in hemolysis buffer, and washed, phosphate buffer solution (PBS) (control group) or 40−60 × 10^^6^ DBA/2 spleen and thymus cells per mouse were injected into the tail vein of B6D2F1 mice at day 0. After 4 weeks, mice were divided into six groups: control, untreated cGVHD, cGVHD treated with high dose LR (2.3 g/kg), cGVHD treated with low dose LR (1.15 g/kg), cGVHD treated with prednisone (5 mg/kg), and cGVHD treated with prednisone (2.5 mg/kg) and LR (2.3 g/kg). The sample size (*n*) for each experimental group was four to thirteen mice as described in the figure legends. Prednisone and LR were dissolved in PBS and administered by daily gavage until the end of the experiment. Control and untreated cGVHD groups received the same dose of PBS.

### 2.4 Dosage of LR and prednisone

LR (Lot: 20190301) was prepared by the Guangdong Second hospital of Traditional Chinese Medicine as dry extract without any additives. Clinically, the daily dosage of this prescription is 92 g/day and the composition was showed at Table 1. The equivalent dosage was 1.15 g/kg/day for mice.^6^ LR was administered at 1.15 and 2.3 g/kg/day, respectively. Prednisone was purchased from Shanghai Sine Pharmaceutical Co., Ltd. The daily dosage of prednisone for mice is 5 mg/kg, as determined by our preliminary experiment that this dosage obtained a best survival of lupus mice (see Supporting Information: Figure S1).

**Table 1:** The component of Lupus Recipe.

Chinese name	Scientific name	Weight(g)
Bai Hua She She Cao	*Scleromitrion diffusum* (Willd.) R.J. Wang (syn.*Hedyotis diffusa* Willd.)	30
Ban Zhi Lian	*Scutellaria barbata* D. Don.	10
Zi Cao	*Lithospermum erytherorhizon* Sieb. Et Zucc.	10
Yi Mu Cao	*Leonurus japonicus* Houtt.	10
Sheng Di Huang	*Rehmannia glutinosa* Libosch.	15
Dan Shen	*Salvia miltiorrhiza* Bunge.	15
Quan Xie	*Scorpion*	2

### 2.5 Western blot

Western blot analysis was performed to detect the protein expression of Rubicon (Rubcn), Nox2, and LC3A/B in the kidney tissues. Briefly, equal amounts of protein samples from kidney cortex fragments were subjected to sodium dodecyl sulfate polyacrylamide gel eletrophoresis (SDS‐PAGE). Immunoreactive bands were visualized using the Shanghai Clinx ChemiScope Touch. The intensity of protein bands was quantified by Image J v1.8.0.

### 2.6 Quantitative real‐time (qRT) **polymerase chain Reaction** (PCR) of renal tissue

Total RNA was extracted from kidney tissues with the TRIzol^TM^ Reagent (Invitrogen, Catalog no. 15596018). The total RNA concentrations were measured by Thermo NanoDrop 2000. complementary DNA (cDNA) was synthesized from total RNA with a Transcriptor First Strand cDNA Synthesis Kit (Roche) according to the manufacturer's instructions. The Synergy Brands (SYBR) green‐based qRT PCR procedure was conducted to amplify the cDNA using the LightCycle480 II (Roche). The primer sequences of target genes were as follows: GAPDH, 5′‐AGGTCGGTGTGAACGGATTTG‐3′ and 5′‐TGTAGACCATGTAGTTGAGGTCA‐3′; and IL‐1β,5′‐GAAATGCCACCTTTTGACAGTG‐3′ and 5′‐TGGATGCTCTCATCAGGACAG‐3′; and IL‐6, 5′‐TAGTCCTTCCTACCCCAATTTCC‐3′ and 5′‐TTGGTCCTTAGCCACTCCTTC‐3′; and IL‐10, 5′‐GCTCTTACTGACTGGCATGAG‐3′ and 5′‐CGCAGCTCTAGGAGCATGTG‐3′; and IFN‐γ, 5′‐ACAGCAAGGCGAAAAAGGATG‐3′ and 5′‐TGGTGGACCACTCGGATGA‐3′; and IFN‐α2, 5′‐TACTCAGCAGACCTTGAACCT‐3′ and 5′‐CAGTCTTGGCAGCAAGTTGAC‐3′; and transforming growth factor‐β (TGF‐β), 5′‐CTCCCGTGGCTTCTAGTGC‐3′ and 5′‐GCCTTAGTTTGGACAGGATCTG‐3′.

### 2.7 Renal histopathology and immunofluorescence

Mice were euthanized after administration for 8 weeks for evaluating the histological scores in the kidneys. After having been fixed in 4% formalin and embedded in paraffin, kidneys were sectioned, and mounted on slides. The kidney tissues of mice were processed for light and immunofluorescent microscopy. The light‐microscopic slides were stained with hematoxylin and eosin (HE) and periodic acid‐Schiff stain (PAS), and they were used to calculate the activity index (AI) and chronicity index (CI) of different groups. The AI and CI were proposed by Austin et al.^17^ and were applied to evaluate semiquantitatively the pathological changes in renal tissues of LN. There are total scores of 24 in AI scores and 12 in CI scores. AI scores are used to evaluate the activity lesions of immune inflammation of renal parenchyma and consist of the following items: endocapillary hypercellularity (score 0−3), glomerular leukocyte infiltration (score 0−3), subendothelial hyaline deposits (score 0−3), fibrinoid necrosis/karyorrhexis (score [0−3] × 2), cellular crescents (score [0−3] × 2) and interstitial inflammation (score 0−3). CI scores are used to measure the chronic irreversible tissue damage of LN and comprised of the following items: glomerular sclerosis (score 0−3), fibrous crescents (score 0−3), tubular atrophy (score 0−3) and interstitial fibrosis (score 0−3). The evaluations were conducted by an experienced renal pathologist. Immunofluorescence slides were stained with IgG (Abcam), blocking with 2% BSA during the overnight incubation of primary antibody at 4°C, observed with a fluorescence microscope (Nikon C2 [Nikon Eclipse Ni‐E]), and the mean fluorescence intensity (MFI) of glomeruli in different groups were calculated using Image J software (NIH). Three glomeruli were randomly selected to calculate the mean MFI of each mouse.

### 2.8 Statistical analysis

Data were expressed as mean ± standard deviation (SD) unless otherwise indicated. Data were analyzed using the unpaired *t*‐test for comparison between two groups or analysis of variance (ANOVA) for comparison among multiple groups as appropriate in GraphPad Prism 7.0. Comparison between two groups in multiple groups used the Bonferroni correction. A two‐way repeated‐measures ANOVA was used for the analysis of proteinuria at different time points. A *p* < .05 was considered statistically significant.

## 3 RESULTS

### 3.1 LR treatment alleviated proteinuria and serum triglyceride levels

**Figure 1:**
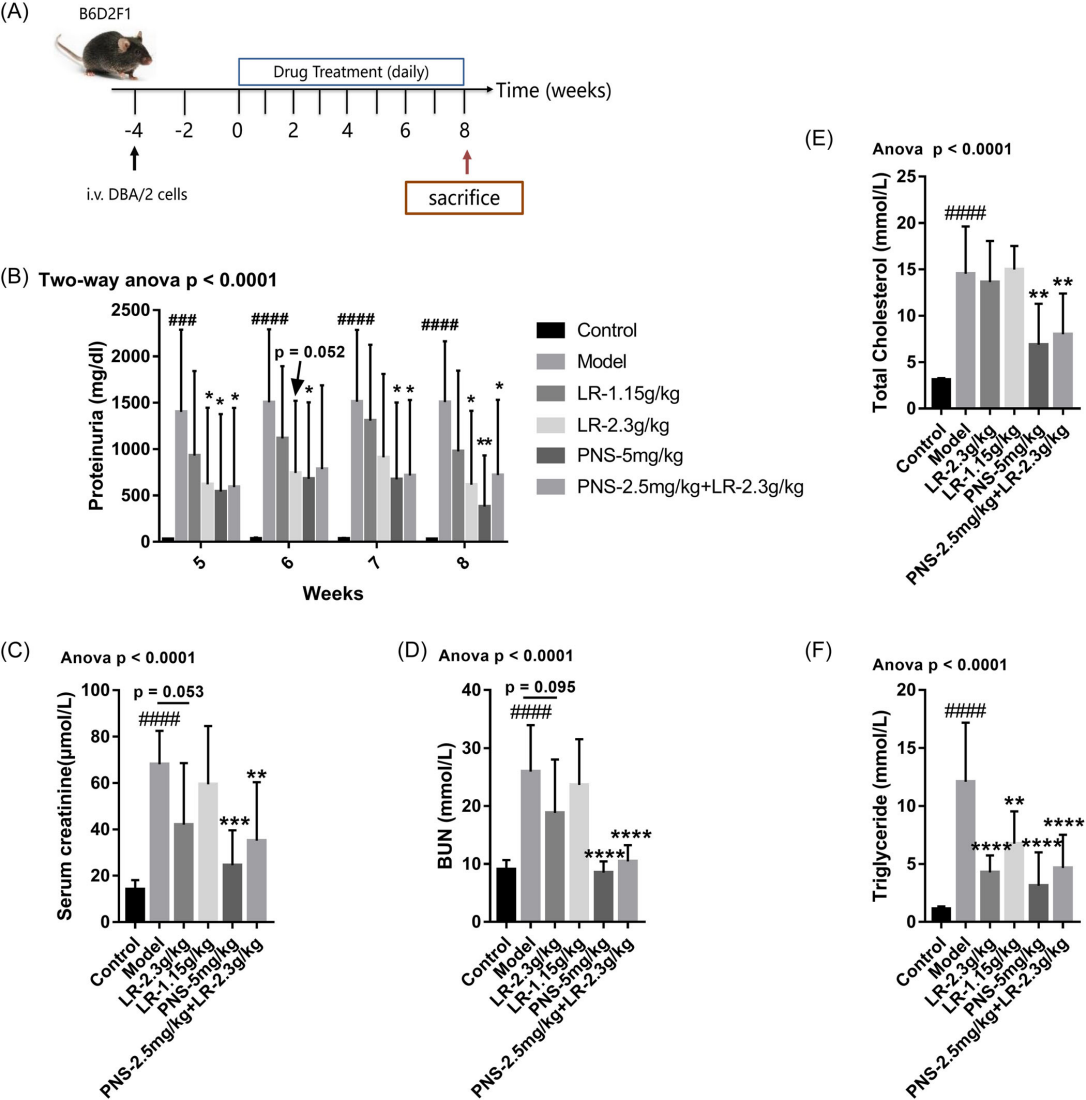
Lupus Recipe or combination treatment reduced proteinuria, serum triglyceride, and serum BUN in the chronic graft versus disease (cGVHD) model. cGVHD model was induced as mentioned in *Materials and Methods*. (A): the experiment design of this study. (B): effect of lupus Recipe or combination treatment on proteinuria after treatment of 5−8 weeks. *p < .05, ***p < .001 versus cGVHD model. *n* = 10−13 per group. (c‐f): effect of different treatment on serum creatinine (Scr) (C), serum blood urea nitrogen (BUN) (D), serum total cholesterol (E), and serum triglyceride (F). Values are expressed as means ± SD; *n* = 8 per group. *p < .05, **p < .01, ***p < .001, ****p < .0001 versus cGVHD model. ##*p* < .01, ###*p* < .001, ####*p* < .0001 versus Control. LR, lupus recipe; PNS, prednisone.

The experimental design of the study was shown in Figure 1A. Proteinuria was a hallmark of LN. In the present study, we showed that LR or combination treatment of LR with low‐dose prednisone (2.5 mg/kg/day) significantly decreased the proteinuria of cGVHD model, as shown in Figure 1B. Treatment with LR at 2.3 g/kg/day slightly decreased the serum creatinine (*p* = .053) and BUN (*p* = .095) after 8 weeks of treatment. While combination treatment of LR with low‐dose prednisone could significantly reduce the serum creatinine (*p* < .01) and BUN (*p* < .0001) (Figure 1C−D). Additionally, the combination treatment of LR with prednisone could also significantly reduce the serum level of total cholesterol (*p* < .01) (Figure 1E) and triglyceride (*p* < .0001) (Figure 1F). Treatment with LR at 2.3 g/kg/day (*p* < .0001) or 1.15 g/kg/day (*p* < .01) also significantly decreased the serum triglyceride (Figure 1F).

### 3.2 LR treatment alleviated the splenomegaly and renal injury

**Figure 2:**
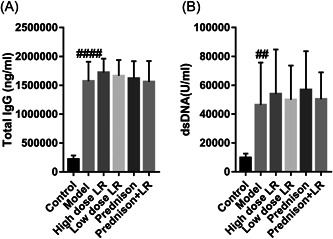
Serum autoantibodies of total IgG (A) and anti‐double‐strained DNA (dsDNA) (B) after treatment at 2 weeks. Values are expressed as means ± SD; *n* = 8 per group. ##*p* < .01, ####*p* < .0001 versus Control. LR, Lupus Recipe; PNS, prednisone.

**Figure 3:**
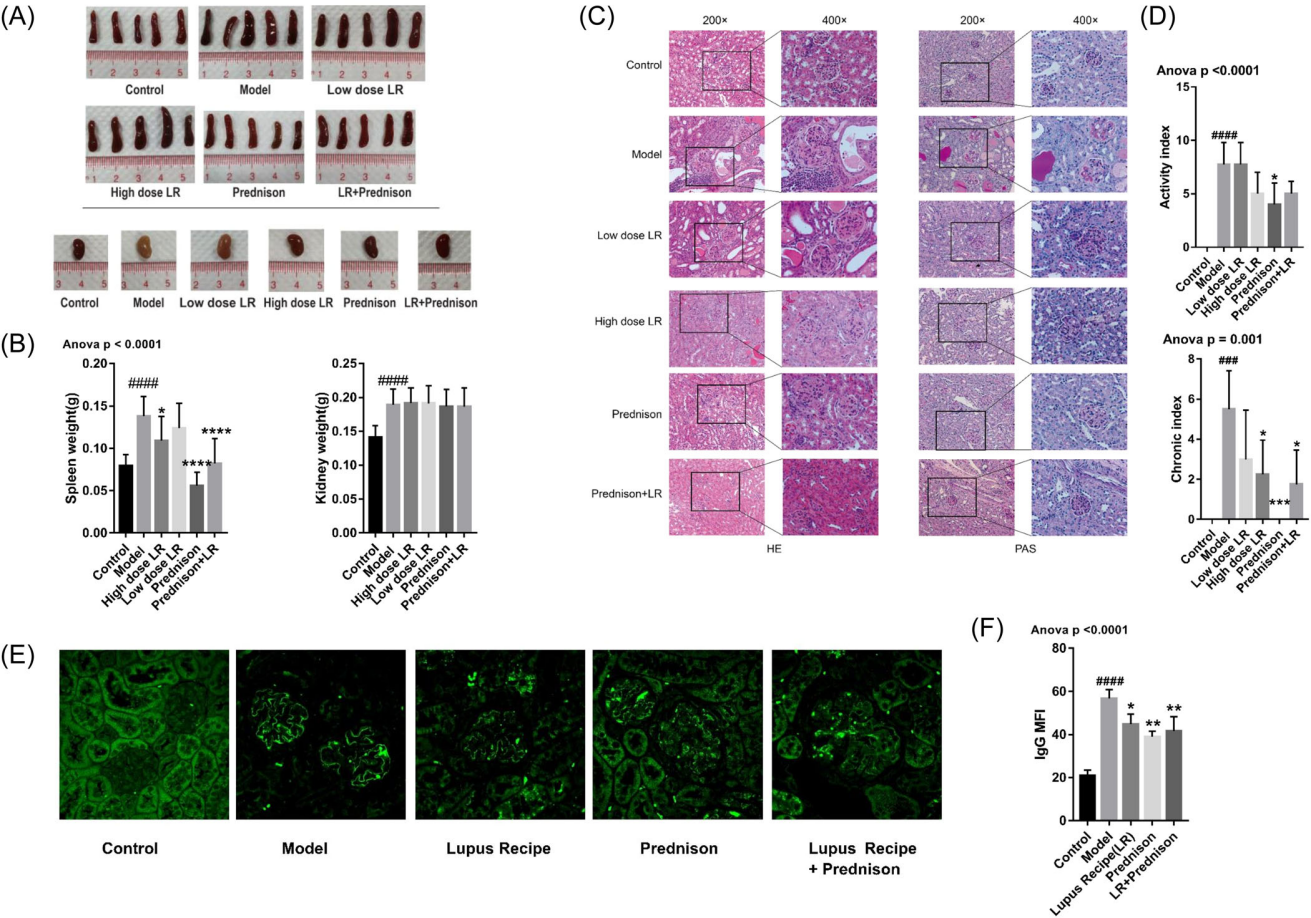
Lupus Recipe or combination treatment alleviated kidney pathology and systemic lupus erythematosus (SLE)‐like phenotypes in the cGVHD model after treatment for 8 weeks. (A): representative spleen and kidney in each group. (B): spleen weight and kidney weight in each group; *n* = 10−13 per group. (C, D): representative renal histopathology in each group (hematoxylin and eosin stain [HE] and periodic acid‐Schiff stain [PAS], 200× and 400×) and comparison between groups; *n* = 4 per group. (E, F): representative renal IgG deposition in each group and comparison between groups; *n* = 3 per group. Values are expressed as means ± SD; ###*p* < .001, ####*p* < .0001versus Control. *p < .05, ****p < .0001versus cGVHD model. LR, Lupus Recipe; MFI, mean fluorescence intensity; PNS, prednisone.

As shown in Figure 2A‐B, the total IgG and dsDNA levels were similar among the different groups after 2‐week treatment. Splenomegaly is a common manifestation of SLE and murine lupus models, and it has been considered a sign of lupus activity. We used spleen weight to evaluate the splenomegaly in this study. As presented in Figure 3A,B, compared to the control mice, the lupus model mice showed an obvious splenic enlargement. After treatment with LR at 2.3 g/kg/day, prednisone, or a combination treatment of LR with prednisone, the splenomegaly was improved significantly. The lupus mice also had a significant increase in kidney weight, but the treatments had no effect on the kidney weight.

Renal pathology was assessed after 8 weeks of treatment. HE and PAS staining showed that substantial protein cast, glomerulosclerosis, and inflammatory cell infiltration appeared in the kidney of lupus mice (Figure 3C). The renal CI score decreased significantly after treatment with LR at 2.3 g/kg/day, prednisone, or a combination treatment of LR with prednisone (Figure 3D). Additionally, our results showed that the LR treatment at 2.3 g/kg/day, prednisone, and combination treatment could significantly decrease IgG deposit in the kidney (Figure 3E,F). The combination treatment of LR with prednisone at 2.5 mg/kg/day showed comparable results in improving renal injury as compared to the treatment with prednisone at 5 mg/kg/day.

### 3.3 LR reduced kidney inflammation

**Figure 4:**
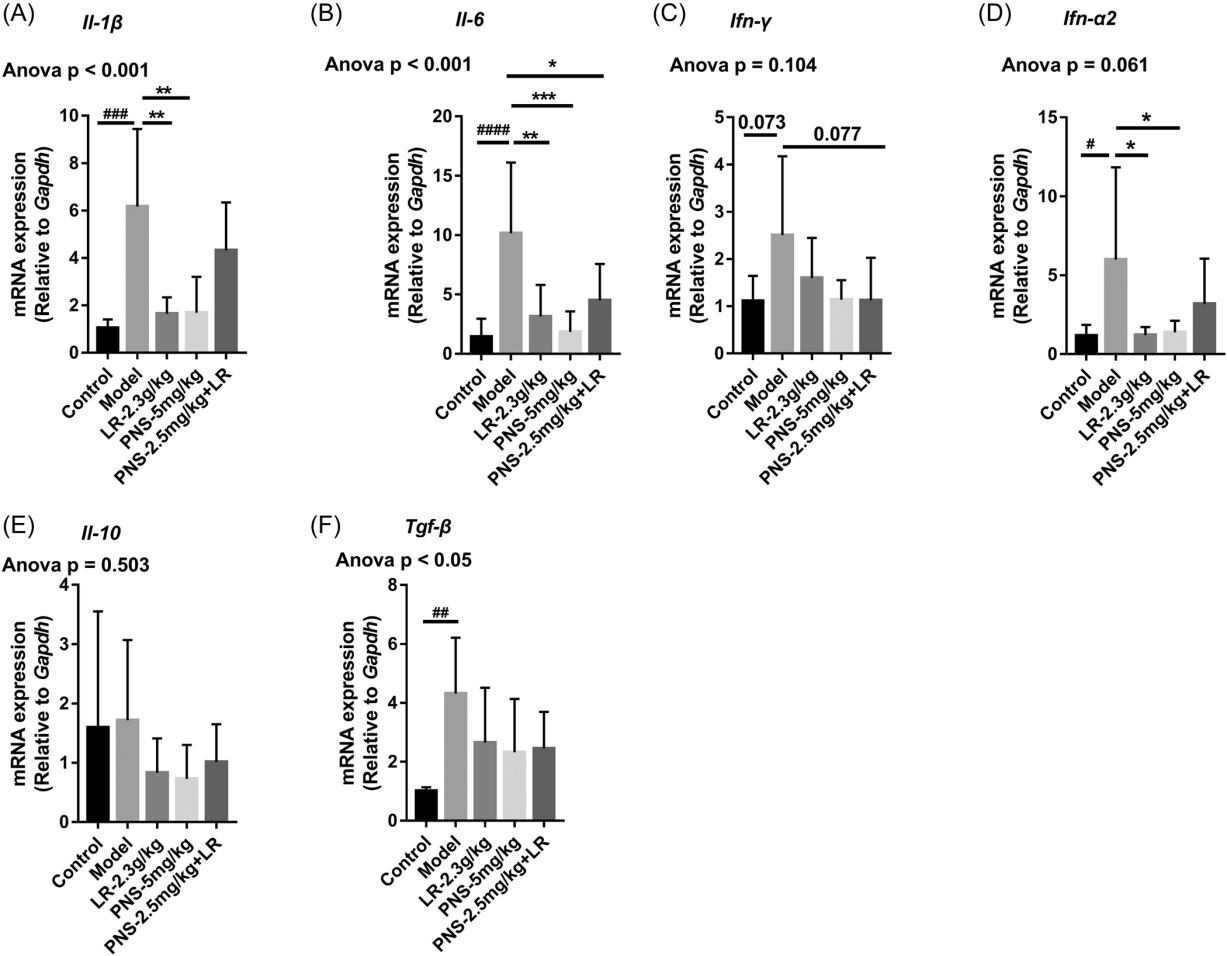
Renal mRNA expression of IL‐1β (A), IL‐6 (B), IFN‐γ (C), IFN‐α2 (D), IL‐10 (E), and TGF‐β (F) (relative to GAPDH). *n* = 4−6 per group. LR, Lupus Recipe; PNS, prednisone.

To evaluate the renal inflammation, we examined the mRNA level of IL‐1β, IL‐6, IFN‐γ, IFN‐α2, IL‐10, and TGF‐β in the kidney. As shown in Figure 4, the model group mice exhibited significantly higher mRNA levels of IL‐1β, IL‐6, and IFN‐α2, and marginally significantly higher mRNA level of IFN‐γ in the kidney tissue, with compared to the control group. Additionally, the model group had a significantly higher mRNA level of TGF‐β than the control group. Compared to the model group, LR treatment at 2.3 g/kg/day could significantly decrease the inflammatory cytokines of IL‐1β, IL‐6, and IFN‐α2 in the kidney. The results were similar to the treatment of prednisone at 5 mg/kg/day. Combination treatment of LR with low‐dose prednisone (2.5 mg/kg/day) inhibited the mRNA expression of IL‐6 and IFN‐γ. The intervention treatments also decreased the mRNA expression of IL‐10 and TGF‐β when compared with the model group, but the differences were not significant.

### 3.4 LR promoted noncanonical autophagy in the kidney

**Figure 5:**
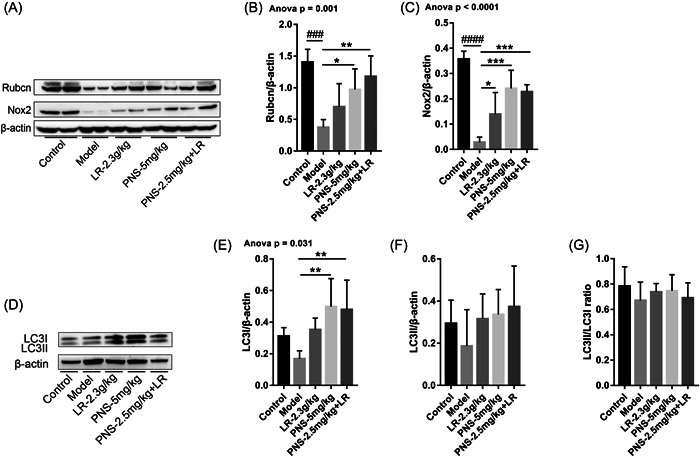
Western blot analysis of LC3‐associated autophagy protein Rubcn and Nox2 (A), quantification analysis of Rubcn relative to β‐actin (B), Nox2 relative to β‐actin (C), western blot analysis of LC3I and LC3II (D), quantification analysis of LC3I relative to β‐actin (E), LC3II relative to β‐actin (F), and ratio of LC3II to LC3I (G). Values are expressed as means ± SD. ### *p* < .001, ####*p* < .0001 versus Control. *p < .05, **p < .001, ***p < .001 versus cGVHD model. *n* = 4 per group. cGVHD, chronic graft versus host disease; LR, Lupus Recipe; PNS, prednisone.

**Figure 6:**
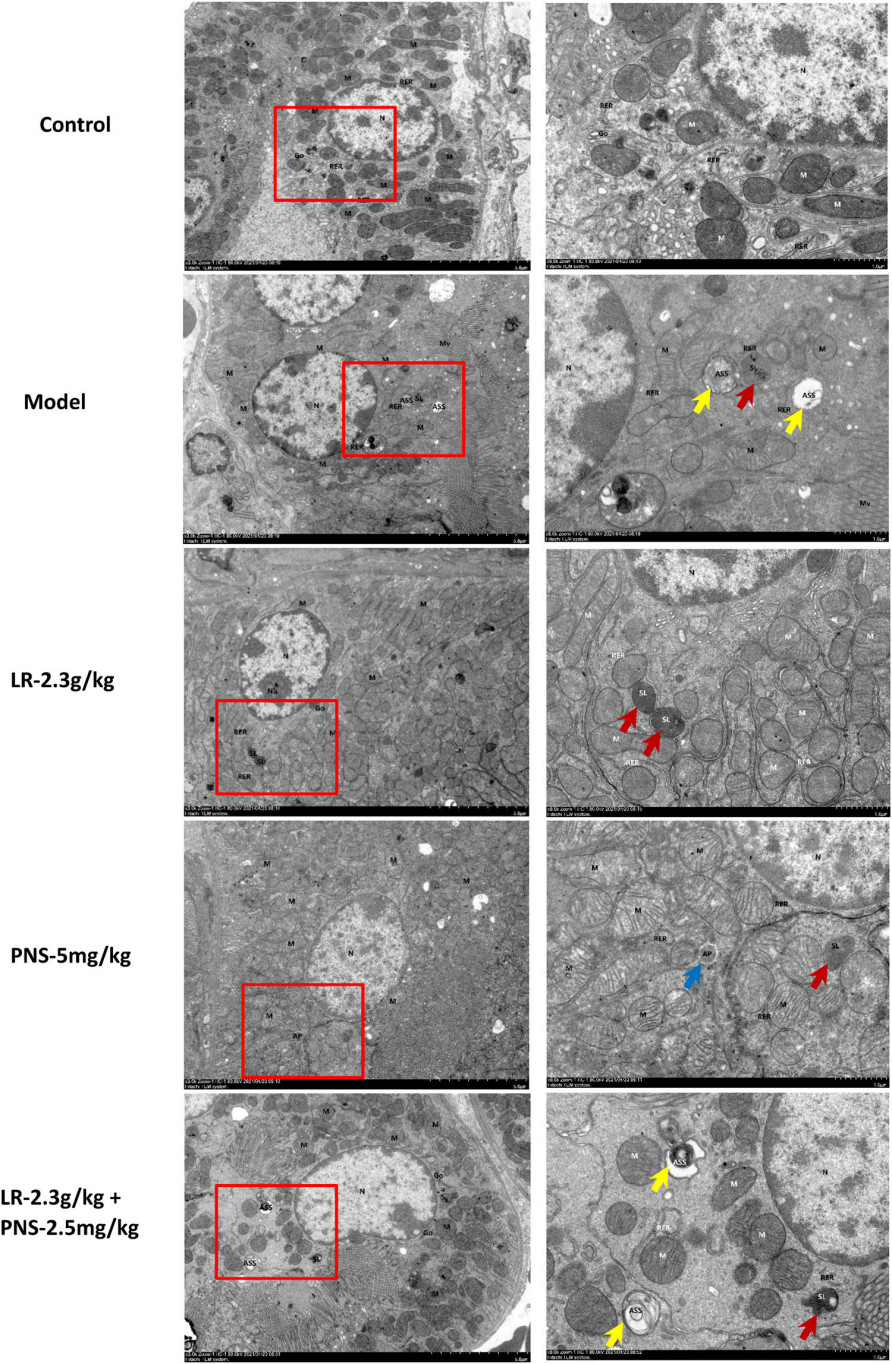
Transmission electron microscopy results of renal tubular epithelial cells in mice with different treatments. AP, autophagosome (blue arrow); ASS, autolysosome (yellow arrow); LR, Lupus Recipe; PNS, prednisone; SL, secondary lysosome (red arrow).

Rubcn and Nox2 are identified as the core component of noncanonical autophagy. In this study, we found that the Rubcn and Nox2 proteins were both significantly decreased in the model group (vs. the control group) in the kidney (Figure 5). With the treatment of LR, the protein expression of Nox2 increased significantly. The expression of Rubcn and LC3‐II in the LR group was slightly higher than the model group, although the difference did not reach significance. With the treatment of LR plus prednisone at 2.5 mg/kg/day, the Rubcn, Nox2, and LC3‐I protein expressions increased significantly, which was the same as that of the prednisone group. In the transmission electron microscopy of renal tubular epithelial cells (Figure 6), we did not observe obvious autophagy in the control group; in the LR treatment group (2.3 g/kg/day), some secondary lysosomes were observed; in the model group, a few secondary lysosomes and autolysosomes were observed; in the prednisone treatment group (5 mg/kg/day), some autophagosomes and secondary lysosomes were found; in the combination group, some secondary lysosomes and autolysosomes were observed.

## 4 DISCUSSION

In this study, our results suggested that LR treatment or combination treatment of LR with prednisone could significantly alleviate the renal damage in cGVHD mice, and the therapeutic effect of LR treatment in combination with a low dose of prednisone (2.5 mg/kg/day) was equal to the high dose of prednisone(5 mg/kg/day). We also found that the LR treatment alone and combination treatment both promoted the LAP process, as indicated by the increased expression of Rubcn, Nox2, and LC3‐II protein levels in renal tissues.

cGVHD model is induced by injection of DBA/2 spleen cells and or thymocytes into female (C57BL/6 × DBA/2) B6D2F1 recipients. In comparison with other lupus models, the cGVHD model is easier to control and can be manipulated to satisfy the needs of researchers. And the variability between individuals is generally decreased. Additionally, it can develop into lupus‐like syndromes rapidly within weeks, while other models take months. And the sex‐bias cGVHD model is similar to the SLE in clinical, in which the female recipients are more susceptible than the male.^18^, ^19^ The above characteristics make the cGVHD model a powerful tool for the study of SLE and LN.

In this herbal prescription of LR, *H. diffusa* Willd and *Scutellaria barbata* Don serve as the principals and are the major component of LR. *S. miltiorrhiza* Bunge and *leonurus japonicus* Houtt serve as the associates and have the effect of activating blood circulation and removing stasis. *H. diffusa* Willd has the function of heat‐clearing and detoxifying, diuresis and dehumidification, and anti‐inflammation.^20^, ^21^ Recent evidence showed that extraction solution from *H. diffusa* Willd had suppressive effects on inflammation and the proliferation of T lymphocytes by regulating the STAT3 signaling pathway, thus ameliorating renal injury in MRL/lpr mice.^22^ Another study proved that *H. diffusa* Willd containing a Chinese Herbal compound could suppress the activation of peritoneal macrophages via modulating the IRAK1‐NF‐κB signaling pathway in MRL/lpr mice.^23^ Baicalein and quercetin are two of the active ingredients of *H. diffusa* Willd and *Scutellaria barbata* Don.^24^ Quercetin could inhibit the activation of CD4 T cells and the release of inflammatory cytokines from macrophages in cGVHD models.^25^ In the pristane‐induced model, quercetin could also ameliorate renal damage and decrease the expression of renal IL‐6, tumor necrosis factor‐α (TNF‐α), and TGF‐β.^26^ Baicalein could improve renal function and renal injury by upregulation of the Nrf2/heme‐oxygenase‐1 signaling pathway in myeloid‐derived suppressor cells of pristane‐induced LN.^27^ Additionally, quercetin could attenuate renal ischemia/reperfusion injury by activating autophagy.^28^

The dysregulation of apoptotic cell clearance due to LAP defects is a critical mechanism for SLE and LN development. The accumulation of apoptotic cells will increase the inflammatory response and promote the disease progression.^13^ In this study, we showed that LAP‐specific Nox2 and Rubicon proteins were both significantly decreased in lupus models, as compared to the control mice. With the treatment of LR or LR combined with a low dose of prednisone, results showed improvement of the renal IgG deposit and chronic injury, and significantly increased expression of LAP‐specific proteins of the kidney, as well as reduced expression of renal inflammatory cytokines and deposition of IgG. These results indicated that the LR treatment might ameliorate the renal injury of lupus mice via modulating the LAP signaling pathway.

As mentioned above, the increased accumulative dosage of glucocorticoid was an important cause of glucocorticoid‐associated damage.^3^, ^4^, ^29^ In the present study, we found that LR treatment in combination with a low dose of prednisone showed a similar result in alleviating renal injury as compared to the high dose of prednisone in lupus mice. These results suggested that the Chinese Medicine LR treatment may be helpful to minimize glucocorticoid usage in SLE and LN treatments.

## 5 CONCLUSION

Collectively, our present study demonstrated that LR treatment could effectively ameliorate the renal damage of lupus mice. And LR treatment might be conducive to reducing the use of glucocorticoids in LN. The mechanism may involve in the regulation of LAP and immune‐inflammatory response.

## AUTHOR CONTRIBUTIONS

**Ruihua Liu**: Data curation (lead); formal analysis (lead); methodology (lead); writing‐original draft (lead); writing‐review and editing (lead). **Xuan Huang**: Data curation (equal); methodology (equal); validation (equal). **Hongjian Ye**: Resources (equal); validation (equal); writing‐original draft (supporting). **Haishan Wu**: Methodology (equal). **Jing Guo**: Methodology (equal). **Yuan Peng**: Methodology (equal). **Meiju Wu**: Methodology (equal). **Jinjin Fan**: Methodology (equal). **Xiao Yang**: Conceptualization (lead); funding acquisition (lead); supervision (lead); writing‐review and editing (equal).

## CONFLICT OF INTEREST STATEMENT

The author declare no conflict of interest.

## ETHICS STATEMENT

The animal study protocol had been reviewed and approved by the Institutional Animal Care and Use Committee (IACUC), Sun Yat‐Sen University.

## Supporting information

Figure S1. Survival rates of lupus mice treated with different dosages of prednisone. PNS, prednisone.

## Data Availability

The data that support the findings of this study are available from the corresponding author upon reasonable request.
