# Supplementary material for: Lupus Recipe inhibits cGVHD‐induced lupus nephritis in mice and promote renal LC3‐associated autophagy
Source: Immun Inflamm Dis. 2023 Mar 17;11(3):e815. doi: 10.1002/iid3.815 (PMC10022419; doi:10.1002/iid3.815)
Supplement: Supplementary file 1 — Figure S1. Survival rates of lupus mice treated with different dosages of prednisone. PNS, prednisone. [file IID3-11-e815-s001.docx]

Supplementary files


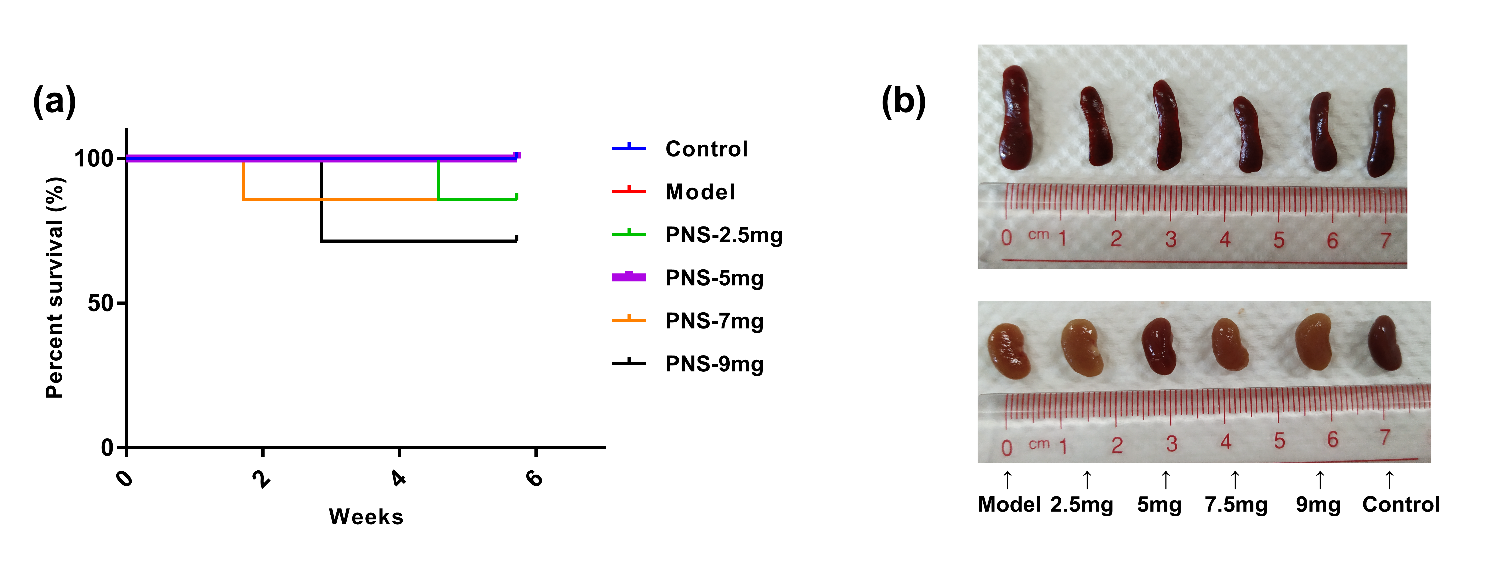


**Figure S1** Survival rates(a) and morphologicchange of kidney or spleen (b) of lupus mice treated with different dosages of prednisone. PNS, prednisone.
